# Jinlida granules ameliorate the high-fat-diet induced liver injury in mice by antagonising hepatocytes pyroptosis

**DOI:** 10.1080/13880209.2022.2029501

**Published:** 2022-02-09

**Authors:** Yuan-yuan Hao, Wen-wen Cui, Huai-lin Gao, Ming-ye Wang, Yan Liu, Cui-ru Li, Yun-long Hou, Zhen-hua Jia

**Affiliations:** a College of Integrative Medicine, Hebei University of Chinese Medicine, Hebei, China; b Hebei Yiling Pharmaceutical Research Institute, Hebei, China; c Hebei Yiling Hospital, Hebei, China; d Xianghe Hospital of Traditional Chinese Medicine, Hebei, China; e National Key Laboratory of Luobing Research and Innovative Chinese Medicine, Hebei, China

**Keywords:** NAFL, traditional Chinese medicine, pyroptosis pathway, HepG2

## Abstract

**Context:**

Jinlida (JLD) as a traditional Chinese medicine formula has been used to treat type 2 diabetes mellitus (T2DM) and studies have shown its anti-obesity effect.

**Objective:**

To investigate the therapeutic effects of JLD in a mouse model of non-alcoholic fatty liver (NAFL).

**Materials and methods:**

C57BL/6J mice were divided into three groups and fed a low-diet diet (LFD), high-fat diet (HFD), or HFD + JLD (3.8 g/kg) for 16 weeks, respectively. The free fatty acids-induced lipotoxicity in HepG2 cells were used to evaluate the anti-pyroptotic effects of JLD. The pharmacological effects of JLD on NAFL were investigated by pathological examination, intraperitoneal glucose and insulin tolerance tests, western blotting, and quantitative real-time PCR.

**Results:**

*In vivo* studies showed that JLD ameliorated HFD-induced liver injury, significantly decreased body weight and enhanced insulin sensitivity and improved glucose tolerance. Furthermore, JLD suppressed both the mRNA expression of caspase-1 (1.58 vs. 2.90), IL-1β (0.93 vs. 3.44) and IL-18 (1.34 vs. 1.60) and protein expression of NLRP3 (2.04 vs. 5.71), pro-caspase-1 (2.68 vs. 4.92) and IL-1β (1.61 vs. 2.60). *In vitro*, JLD inhibited the formation of lipid droplets induced by 2 mM FFA (IC_50_ = 2.727 mM), reduced the protein expression of NLRP3 (0.74 vs. 2.27), caspase-1 (0.57 vs. 2.68), p20 (1.67 vs. 3.33), and IL-1β (1.44 vs. 2.41), and lowered the ratio of *p*-IKB-α/IKB-α (0.47 vs. 2.19).

**Conclusion:**

JLD has a protective effect against NAFLD, which may be related to its anti-pyroptosis, suggesting that JLD has the potential as a novel agent in the treatment of NAFLD.

## Introduction

Non-alcoholic fatty liver disease (NAFLD) has become one of the most prevalent chronic liver diseases along with the obesity epidemic and sedentary lifestyles (Chan et al. 2019). The global prevalence of NAFLD is approaching 25.24% with the highest prevalence in the Middle East and South America and lowest in Africa (Lonardo et al. 2016). Recent data show that the prevalence of NAFLD in China dramatically increased from 18% to 29% over the past 10 years (Fan et al. 2017). The chronic pathological process of NAFLD spans from the hepatic steatosis caused by triglyceride accumulation, classified histologically as the NAFL, to the steatohepatitis (NASH) with varying degrees of liver fibrosis (Arab et al. 2018). Due to the deterioration of liver function, NAFLD causes systemic metabolic abnormalities such as obesity, dyslipidemia, and T2DM (Anstee et al. 2013; Mao et al. 2019). More seriously, NAFLD may eventually lead to hepatocellular carcinoma (HCC) (Younossi et al. 2015). Thus, we are facing challenges from this serious global health problem. Unfortunately, in addition to the diet and exercise control, there are no FDA-approved potent medicines for NAFLD so far (Chen et al. 2019). Therefore, there is an urgent need to develop effective pharmacological treatments to combat NAFLD.

NAFL and NASH represent two different clinicopathological entities in NAFLD, respectively. The liver biopsy data suggest that NAFL can progress to NASH (Mcpherson et al. 2015), and the significant hepatocyte injury and dysfunction occur in the NASH stage, characterised by hepatocellular death, inflammation, and fibrosis. Although the progression of NASH is multifactorial and its understanding is still incomplete, the fibrosis deposition suggests that the hepatic lipotoxicity leads to local inflammation, and an inflammation-induced activation of cell death pathways may coordinate the lipotoxicity to aggravate hepatic injuries (Arab et al. 2018). Thus, the inflammation-induced cell death pathway is closely associated with the development and progression of NAFLD. Pyroptosis, as the inflammation-dependent form of cell death, is mediated by the activation of NOD-like receptor family pyrin domain-containing 3 (NLRP3) inflammasomes (Gaul et al. 2021). Animal models of NASH have demonstrated the inactivation of NLRP3 inflammasome can inhibit hepatocyte pyroptosis and protect mice from HFD-induced NASH (Blasetti Fantauzzi et al. 2017), which suggests that NLRP3 may represent a novel therapeutic target.

Jinlida granule is an approved patent medicine by the National Medical Product Administration (NMPA) and is listed in the Chinese pharmacopoeia and National Directory of Health Insurance. The approval number is Z20050845, and it consists of 17 herbal medicines. The compound herbs in JLD are designed on the basis of the traditional Chinese medicine (TCM) theory-spleen deficiency for the treatment of T2DM with Qi and Yin deficiency symptoms, either as monotherapy or as an adjunct therapy (Lian et al. 2015), shown in Table 1. According to the combination principles, *Ginseng Radix Et Rhizoma* works as the monarch drug in JLD prescription, and ginsenoside Rb1, ginsenoside CK, and polysaccharides contained in the *Ginseng Radix Et Rhizoma* have been reported to exert an anti-hyperglycemic effect and stimulate the glucose uptake by adipocytes (Huang et al. 2010; Sun et al. 2014; Zhou et al. 2019). The *Sophorae flavescentis Radix* functioned as the ministerial drug, and matrine, as an active ingredient, not only has therapeutic effects against type 2 diabetes (T2D) (Guo et al. 2014), but also shows great potential in treating hepatosteatosis induced by high-fructose and high-fat diets (Liu et al. 2017; Mahzari et al. 2018). *Polygonati Rhizoma* and *Atractylodis Rhizoma* have a wide range of pharmacological activities, including anti-inflammatory, antioxidant, and antidiabetic activities (Deng et al. 2012; Wang et al. 2017; Lyu et al. 2019). JLD has been reported to improve HFD-induced metabolic disorders such as hepatic steatosis, dyslipidemia, insulin resistance, hyperglycaemia, and obesity (Zhang et al. 2019). JLD improves liver damage in diabetic rats and ameliorates liver oxidative stress in high-fat-fed rats (Liu et al. 2015). The molecular mechanisms of metabolic improvements indicate that JLD increases the expression of genes involved in mitochondrial function and fat oxidation to reduce intracellular lipid accumulation (Wang et al. 2018), and JLD activates brown adipose tissue (BAT) thermogenesis through the improvement of mitochondrial biogenesis and fatty acid oxidation metabolism (Zhang et al. 2019).

To summarise, animal studies prove that JLD is a potent TCM in lowering glucose and regulating metabolism and reveal its multi-target roles in the treatment of metabolic disorders. Recently, a 12-week randomised placebo-controlled double-blind multicenter study with 192 T2DM patients (186 completed the study) corroborates the efficacy of JLD in metabolic disorders and potential mechanisms under clinical evidence (Tian et al. 2018).

**Table 1.** Complex compounds contained in JLD.

Plants	Amount in application (ratio)
*Panax ginseng* C. A. Meyer. [Araliaceae; Panax]	10
*Polygonatum kingianum* Coll. et Hemsl. [Liliaceae; Polygonatum]	12
*Atractylodes lancea (Thunb.)* DC. [Compositae; Atractylodes]	6
*Sophora flavescens* Ait. [Leguminosae; Sophora]	5
*Ophiopogon japonicus* (Linn. f.) Ker-Gawl. [Liliaceae; Ophiopogon]	12
*Rehmanniag lutinosa* Libosch. [Scrophulariacease; Rehmannia]	9
*Polygonum multiflorum* Thunb. [Polygonaceae; Fallopia]	8
*Cornus officinalis* Sieb. et Zucc. [Cornaceae; Cornus]	12
*Poria cocos* (Schw.) Wolf. [Polyporaceae; Poria Pers.ex Grag.]	8
*Eupatorium fortunei* Turcz. [Compositae; Eupatorium]	5
*Coptis chinensis* Franch. [Ranunculaceae; Coptis]	5
*Anemarrhena asphodeloides* Bunge. [Liliaceae; Anemarrhena]	6
*Epimedium brevicornu* Maxim. [Berberidaceae; Epimedium]	5
*Salvia miltiorrhiza* Bunge. [Labiatae; Salvia]	8
*Lycium chinense* Mill. [Solanaceae; Lycium]	8
*Pueraria lobata* (Willd.) Ohwi. [Leguminosae; Pueraria]	12
*Litchi chinensis* Sonn. [Sapindaceae; Litchi]	12

The active compounds in the JLD mainly include salvianic acid A sodium, puerarin, salvianolic acid B, epimedin B, epimedin C, icariin, ginsenoside Rb1, ginsenoside Rc, and ginsenoside Rb2.

In the present study, we elucidated the protective effects of JLD on HFD induced hepatocyte pyroptosis via inactivation of the NLRP3 pathway, and the consequent inflammatory responses in a NAFLD mouse model and cultured hepatocytes.

## Materials and methods

### Preparation of Jinlida

JLD granules were obtained from Shijiazhuang Yiling Pharmaceutical Co., Ltd. (Shijiazhuang, China, batch number S-1310001). UPLC fingerprints of Jinlida granules consist of 50 common peaks, and 9 of these common peaks are identified (referred to as previously reported) (Wang et al. 2015). In animal experiments, JLD was dissolved in 0.5% carboxymethyl cellulose (CMC-Na) solution and administered at a dose of 3.8 g/kg by oral gavage according to the methods reported previously (Liu et al. 2015). After dissolving JLD granules in serum-free Dulbecco’s Modified Eagle Medium (DMEM), the mixture was sonicated and centrifuged at 10 000 *g* for 10 min. After centrifugation, the precipitate was dried at 60 °C, and the supernatant was filtered out (0.22 μm pore size) to calculate an accurate weight of the dissolved ingredients. A stock solution with a final concentration of 100 mg/mL was prepared.

### Animals

All animal experiments (protocol number: 2018028) adhered to the specification of the Animal Care and Use Committee of Hebei Yiling Chinese Medicine Research Institute (Shijiazhuang, China). Fifty male C57BL/6J mice (4 weeks of age, 7–12 g) were purchased from Vital River Laboratory Animal Technology Co. Ltd (Beijing, China) (No.11400700298460) and housed (*n* = 5 per cage) in a standard facility at 22 °C with a 12 h day/night cycle with access to food and drinking water. After a one-week adaptation period, the mice were randomly assigned into 3 groups (*n* = 15): CON + LFD (10 kcal% Fat, D12450B, Research Diets, USA), CON + HFD (60 kcal% Fat, D12492, Research Diets, USA) and HFD + JLD (HFD supplemented with JLD). CMC administration was served as the HFD control treatment. The body weight of each mouse was measured once a week for 16 weeks.

### Liver histochemical staining

After treatment with JLD or CMC for 16 weeks, mice were fasted for 16 h, sacrificed, and the liver was excised and weighed. Liver samples were cut and submerged in 4% paraformaldehyde, embedded in paraffin, sectioned into 6 μm thick sections, and stained with haematoxylin and eosin (H&E). Frozen liver sections were stained with Oil Red O (ORO), as previously described (Derdak et al. 2011).

### Biochemical analysis

The serum levels of total triglyceride (TG), total cholesterol (TC), the low-density lipoprotein cholesterol (LDL-C), the high-density lipoprotein cholesterol (HDL-C), non-esterified fatty acids (NEFA), aspartate aminotransferase (AST), and alanine aminotransferase (ALT) were determined by a commercial kit (Jiancheng Institute of Biotechnology, Nanjing, China) according to the manufacturer’s protocols. These indicators were measured on an automated biochemical analyser (Hitachi 7080, Japan).

### Intraperitoneal glucose tolerance test (IPGTT) and insulin tolerance test (ITT)

Blood samples were taken from the tail vein for intraperitoneal glucose tolerance test (IPGTT) and insulin tolerance test (ITT) at the 14^th^ week. Blood glucose levels were measured using an automatic glucose metre (Roche Diagnostics, Germany). For the IPGTT, mice fasted for 16 h and then received an intraperitoneal injection of glucose (1.5 g/kg). The mice's blood glucose levels were monitored at 0, 30, 60, 90, and 120 min. For the ITT, the mice were fasted for 6 h and intraperitoneally injected with recombinant human insulin (0.8 U/kg). The blood glucose levels of the mice were monitored at 0, 15, 30, 60, and 90 min.

### Cell culture and treatments

HepG2 cells were obtained from the American Type Culture Collection (ATCC; Manassas, VA, USA). Cells were cultured in Dulbecco’s Modified Eagle’s Medium (DMEM) (Gibco, 12100-046, USA) supplemented with 15% foetal bovine serum (FBS) (Gibco Invitrogen Corporation, USA), and 1% penicillin/streptomycin (P/S) in an incubator with 5% CO_2_ at 37 °C. Free fatty acids (FFAs; oleic acid and palmitic acid, 2:1) were dissolved in isopropyl alcohol. Hepatic steatosis induced by FFA (BSA, Solarbio) was used as a non-alcoholic fatty liver model *in vitro*. (Du et al. 2017; Schmidt et al. 2017; Zhao et al. 2018).

HepG2 cells were seeded at a density of 1 × 10^5^ cells/well in a 96-well plate and allowed to adhere to the wells. For induction of hepatic steatosis, HepG2 cells were incubated with FFA (0, 1, 2, 3, 4 mM) in a serum-free medium containing 1% BSA to stimulate lipid accumulation for 24 h. Then, cells were incubated in serum-free DEME containing 2 mM of FFA, with or without JLD treatment at different doses for 24 h. Then, MTS reagent (5 mg/mL, G3581, Promega) was added for another coincubation of 2 h. Finally, the absorbance was determined at 490 nm and 630 nm with a microplate reader (BioTek). The HepG2 cells were divided into three groups: (i) control group, HepG2 were cultured in a serum-free medium; (ii) FFA group, HepG2 were cultured in FFA solution (2 mM); and (iii) FFA + JLD group, HepG2 were pre-treated with JLD (200 µg/mL).

HepG2 cells (1 × 10^5^ cells/well) were seeded in 6-well plates and allowed to adhere to the wells. Then, cells were incubated in DMEM containing 2 mM of FFA, with JLD treatment at different doses for 24 h. Then, intracellular TG and AST levels were detected by a commercial kit (Jiancheng Institute of Biotechnology, Nanjing, China).

### Nile red staining

The lipid accumulation of HepG2 cells was examined by staining with the lipophilic dye Nile Red (Sigma-Aldrich). Nile red was added to a final concentration of 30 μM and incubated at 37 °C for 10 min. The average fluorescence intensity of the lipid droplets was observed using a high-intensity microscope (Operetta).

### Quantitative real-time PCR analysis (qRT-PCR)

The total RNAs of HepG2 cells and the excised liver were extracted with the Eastep TM total RNA extraction kit (Promega, China). The purity and concentration of RNA were determined using a micro nucleic acid protein detector (Gene 2000, Thermo) and samples with A260/A280 values ranging from 1.9 to 2.1 were selected. Reverse transcription was performed according to the instructions of Prime Script TM Synthesis Kit (Takara, China) to obtain cDNA, and the real-time fluorescent quantitative PCR reaction was carried out in the ABI 7900 H sequence detection PCR system according to the SYBR Green PCR Master Mix (Takara, China) kit instructions. The sequence of primers in the experiments was listed in Table 2.

**Table 2.** Primer sequences used for qRT-PCR in this study.

Primer	Forward	Reverse
Caspase1	CGTCTTGCCCTCATTATCTGC	CTGTCAGAAGTCTTGTGCTCT
IL-1β	GCAACTGTTCCTGAACTCAACT	ATCTTTTGGGGTCCGTCAACT
IL-18	GACTCTTGCGTCAACTTCAAGG	CAGGCTGTCTTTTGTCAACGA

### Western blotting analysis

Protein concentration of HepG2 and liver tissue were determined with a BCA protein assay kit before electrophoresis of 4-20% SDS-PAGE. Proteins were then transferred onto a Nitrocellulose Blotting membrane (Pall, USA). The membranes were blocked using Odyssey blocking Buffer for 1 h and incubated with the primary antibodies specific for NLRP3 (15101, CST; 19771, Proteintech), caspase-1 (3866, CST; 22915, Proteintech), IL-1β (2105, Abcam), *p*-IKB-α (133462, Abcam), IKB-α (76429, Abcam) and GAPDH (5174, CST) overnight at 4 °C. The primary antibodies were removed by washing the membranes three times in Tris Buffered Saline (TBS) (G0001, Servicebio), 10 min each. Membranes were incubated in secondary antibodies (Dnk pAb to Rb IgG IRDye 680RD, 216779; Goat pAb to Ms IgG IRDye 800CW, 216772, Abcam) for 1 h at room temperature. Signals were detected by the Odyssey imaging system and quantified by image analysis software (LI-COR, USA).

### Statistical analysis

All data were statistically analysed using SPSS 23.0 statistical software. The data were expressed as mean ± SD. For multiple comparisons, a one-way analysis of variance (ANOVA) was used. The LSD method or nonparametric statistics test was used for pairwise comparison. Differences were considered statistically significant when the *p*-value was less than 0.05.

## Results

### JLD protected mice from HFD-induced body weight gain and insulin resistance

**Figure 1.**
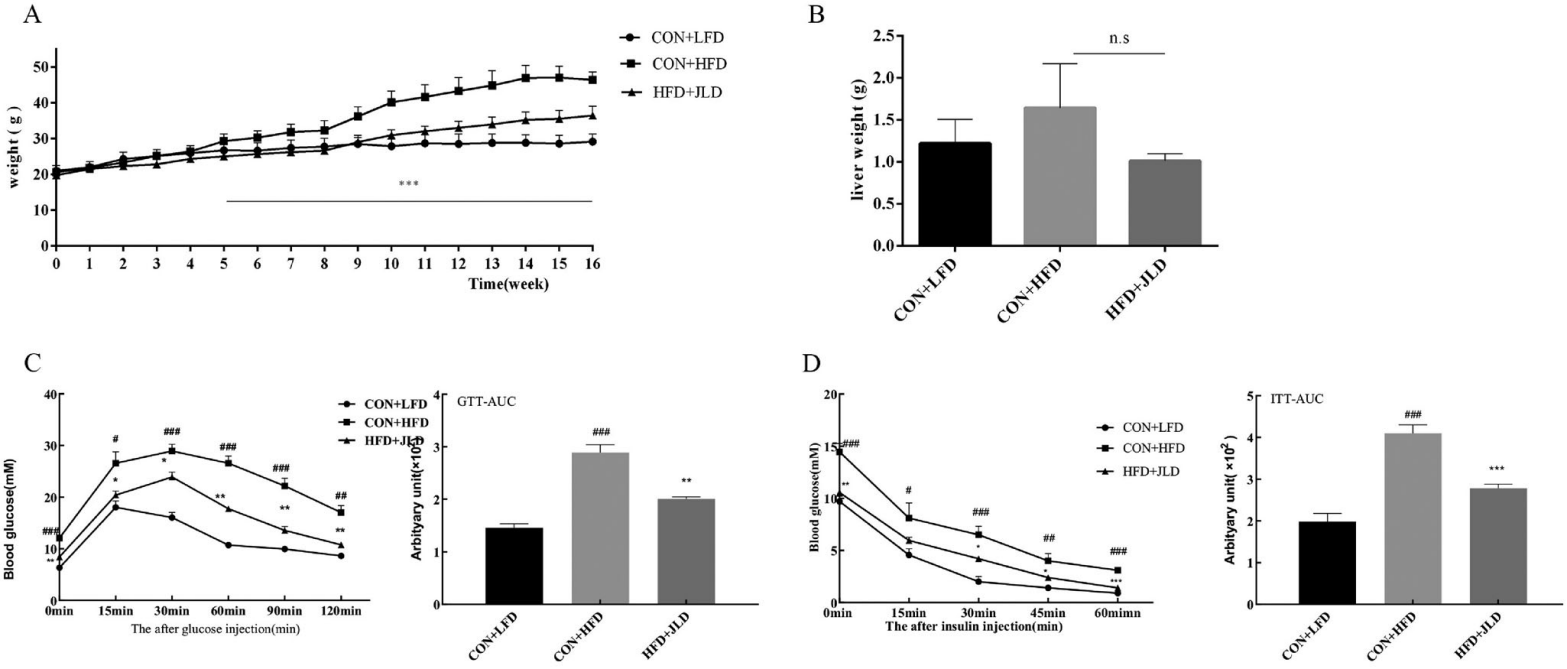
JLD reduced body weight gain and blood glucose in HFD-induced obese mice. (A) Weight-time curve after 16 weeks of intervention (*n* = 15). (B) Liver weight (*n* = 6). (C) Time-dependent blood glucose concentrations during the IPGTT test and IPGTT-AUC (*n* = 8). (D) Time-dependent blood glucose concentrations during the ITT test and ITT-AUC (*n* = 8). Data are shown as the mean ± SD. n.s, nonsignificant. #*p* < 0.05, ##*p* < 0.01, ###*p* < 0.001 vs. CON + LFD. *p < 0.05, **p < 0.01, ***p < 0.001 vs. CON + HFD.

The effects of JLD on the bodyweight of mice fed with a high-fat diet (HFD) during 16 weeks of treatment are shown in Figure 1(A). The bodyweight of mice fed with an HFD increased significantly from the 5^th^ week compared with mice treated with JLD until the end of the experiment (*p* < 0.001). Interestingly, there was no significant difference in the weights of livers excised from HFD mice fed with or without JLD (Figure 1(B)). Glucose tolerance tests and insulin tolerance tests showed that JLD enhance insulin sensitivity and improved glucose tolerance (Figure 1(C,D)) (*p* < 0.01, *p* < 0.001).

### JLD improved lipid accumulation

**Figure 2.**
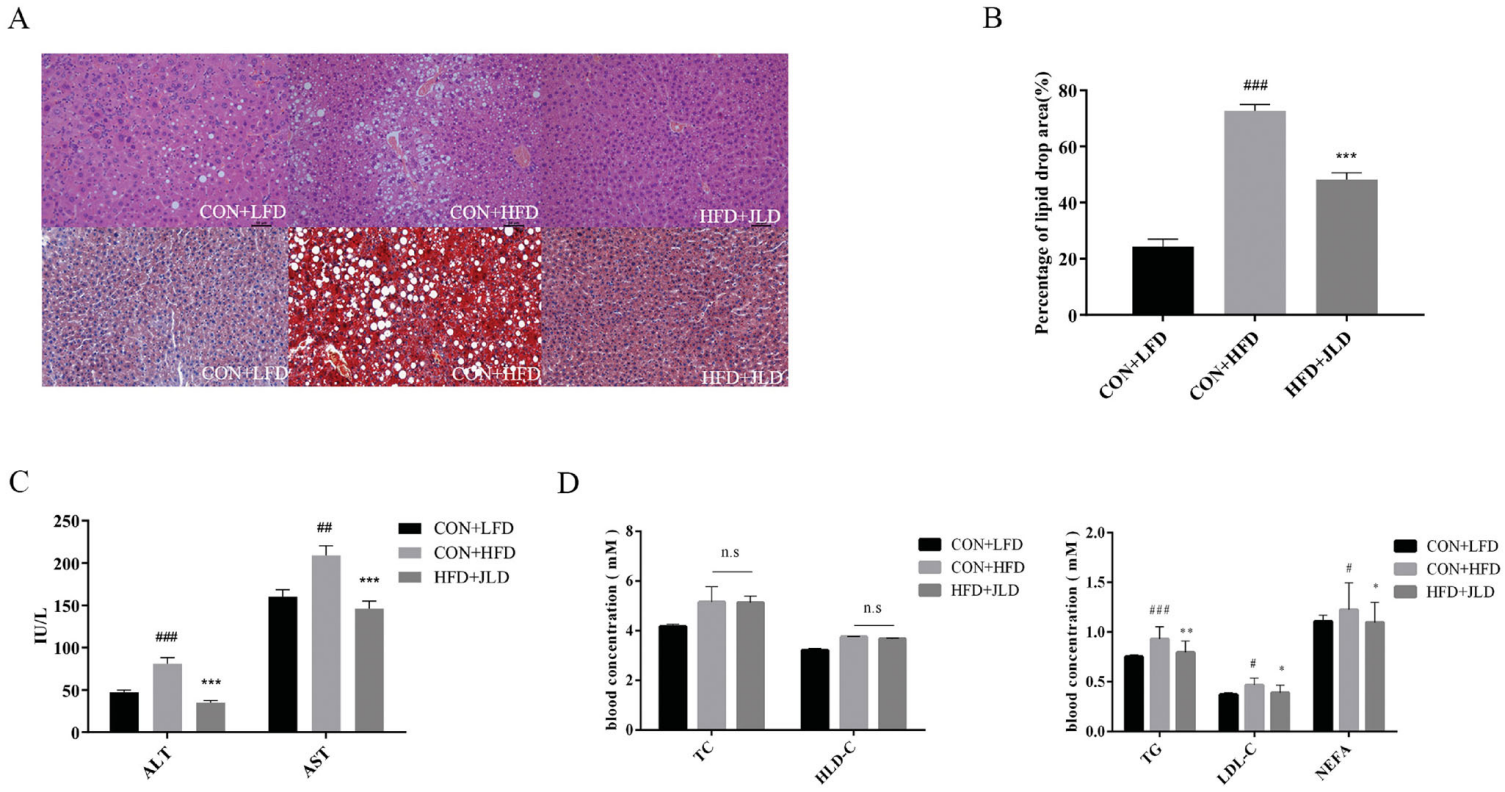
JLD maintained lipid homeostasis in HFD-fed obese mice. (A) Representative H&E staining, and ORO staining of liver (200×). (B) Percentage of lipid drop area (%) (C–E) Fasting serum lipid levels of ALT, AST, TG, LDL-C, NEFA, TC and HDL-C (*n* = 8). Data are shown as the mean ± SD. n.s, nonsignificant. #*p* < 0.05, ##*p* < 0.01, ###*p* < 0.001 vs. CON + LFD. *p < 0.05, **p < 0.01, ***p < 0.001 vs. CON + HFD.

By hepatic H&E and Oil Red O staining, we found that HFD-fed mice had severe lipid accumulation and inflammation. Notably, JLD significantly reduced the number of lipid droplets and inflammatory cell infiltration (Figure 2(A)). JLD significantly reduced the percentage of lipid droplet areas (Figure 2(B)) (*p* < 0.001). We also assessed the serum levels of mice fed the HFD diet and found an elevation in the serum ALT and AST levels. However, when mice fed the HFD diet were treated with JLD, a reduction in serum ALT and AST levels was observed (Figure 2(C)). Compared with the HFD group, JLD significantly reduced TG, LDL-C, and NEFA levels in serum (Figure 2(D,E)) (*p* < 0.05, *p* < 0.01, *p* < 0.001).

### JLD inhibited pyroptosis

**Figure 3.**
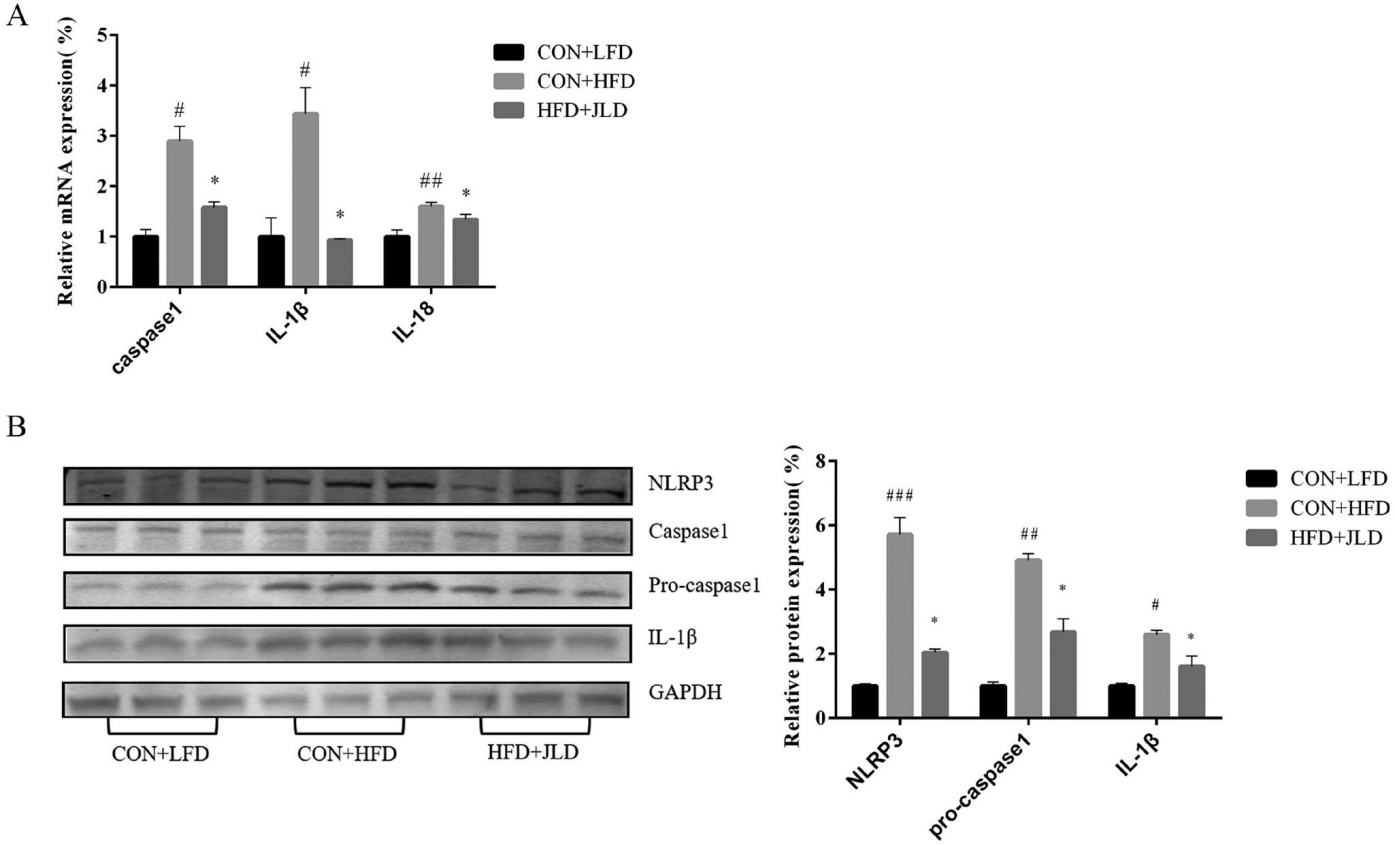
JLD ameliorated HFD-induced pyroptosis in mice. (A) qRT-PCR analysis of proptosis-related gene caspase 1, IL-1β and IL-18 expression in liver (*n* = 3). (B) Western blot analysis of liver proptosis-related protein NLRP3, pro-caspase 1 and IL-1β expression in liver (*n* = 3). Data were normalised to GAPDH for western blot. Data are shown as the mean ± SD. #*p* < 0.05, ##*p* < 0.01, ###*p* < 0.001 vs. CON + LFD. *p < 0.05 vs. CON + HFD.

The mRNA expression of caspase1, IL-1β and IL-18 increased by 2.89-, 3.44-, and 1.6-fold, respectively (*p* < 0.05, *p* < 0.01). At the same time, the protein expression of NLRP3, pro-caspase-1, and IL-1β also increased (*p* < 0.05, *p* < 0.01, *p* < 0.001), indicating the induction of pyroptosis in HFD-fed mice. In the JLD group, the expression of these genes and protein significantly decreased (*p* < 0.05, *p* < 0.01, *p* < 0.001) (Figure 3(A,B)).

### JLD improved lipid accumulation and biochemical parameters *in vitro*

**Figure 4.**
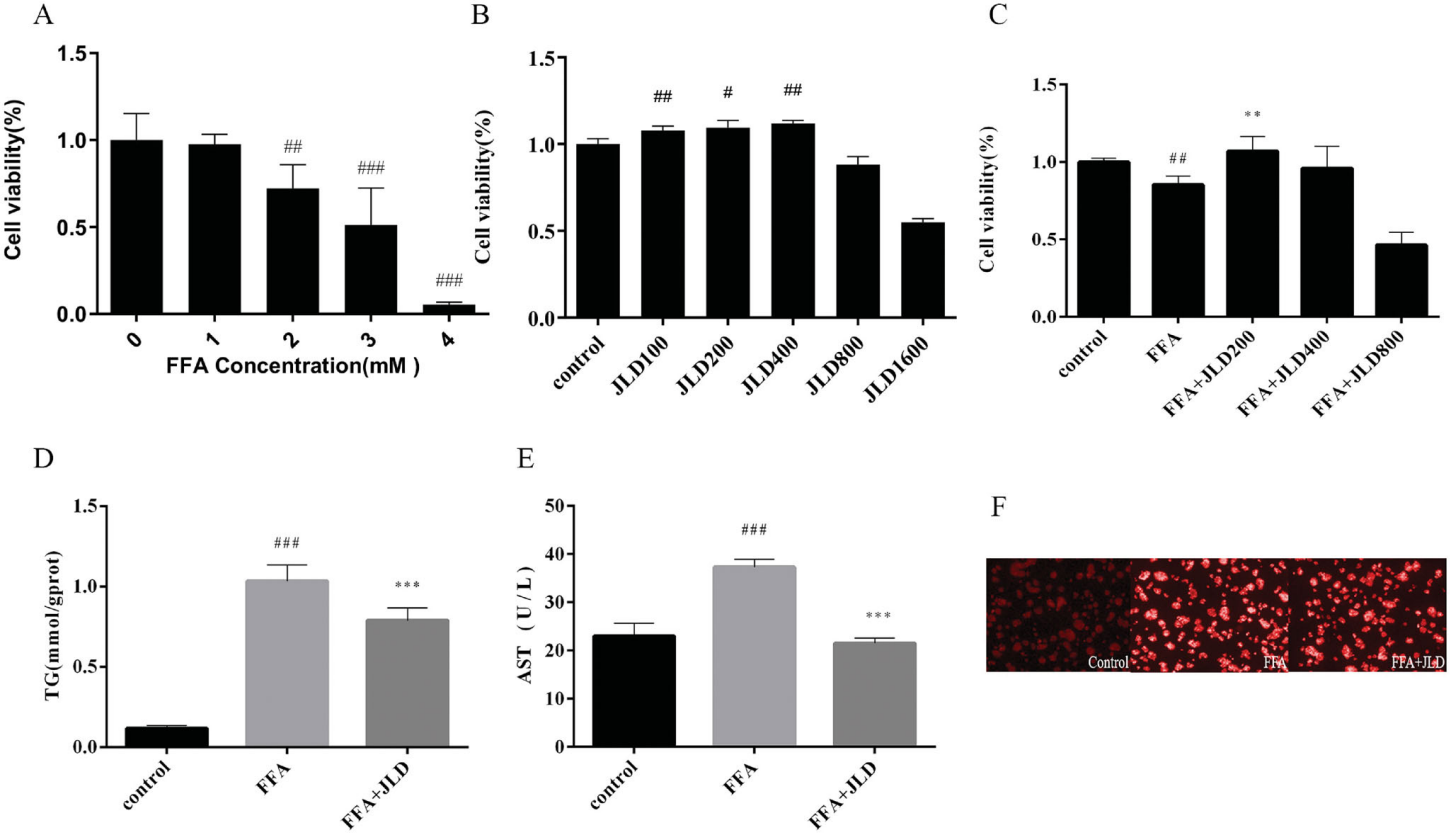
Effects of JLD treatment on steatosis cell model. (A) Cell viability treated with FFA; (B) Cell viability treated with JLD; (C) Effects of different concentrations of JLD on lipid decrease in HepG2 cells. (D,E) Effects of JLD on TG and AST accumulation in HepG2 cells. (F) HepG2 cell Nile red staining. Data are shown as the mean ± SD. #*p* < 0.05, ##*p* < 0.01, ###*p* < 0.001 vs. CON. *p < 0.05, **p < 0.01, ***p < 0.001 vs. FFA

The effect of FFA and JLD on the viability of HepG2 cells was assessed. As shown in Figure 4(A–C), 2 mM FFA reduced cell viability to approximately 70%, and concentrations of 100, 200, and 400 μg/mL JLD were not cytotoxic to HepG2 cells (*p* < 0.05, *p* < 0.01, *p* < 0.001). The calculated IC_50_ value was 2.727 mM. Thus, FFA (2 mM) and co-treatment with JLD (200 μg/mL) was used to evaluate drug effects in HepG2 cells in this study (*p* < 0.01). Simultaneously, we also evaluated the effects of JLD on TG and AST, and the results showed that JLD significantly reduced the content of TG and AST (Figure 4(D,E)) (*p* < 0.001). Compared with the control group, Nile red staining showed extensive lipid vacuoles in the FFA group of HepG2 cells. JLD treatment significantly reduced cellular lipid deposition in HepG2 cells, indicating its protective role in preventing lipid accumulation (Figure 4(F)).

### JLD improved FFA-induced pyroptosis

**Figure 5.**
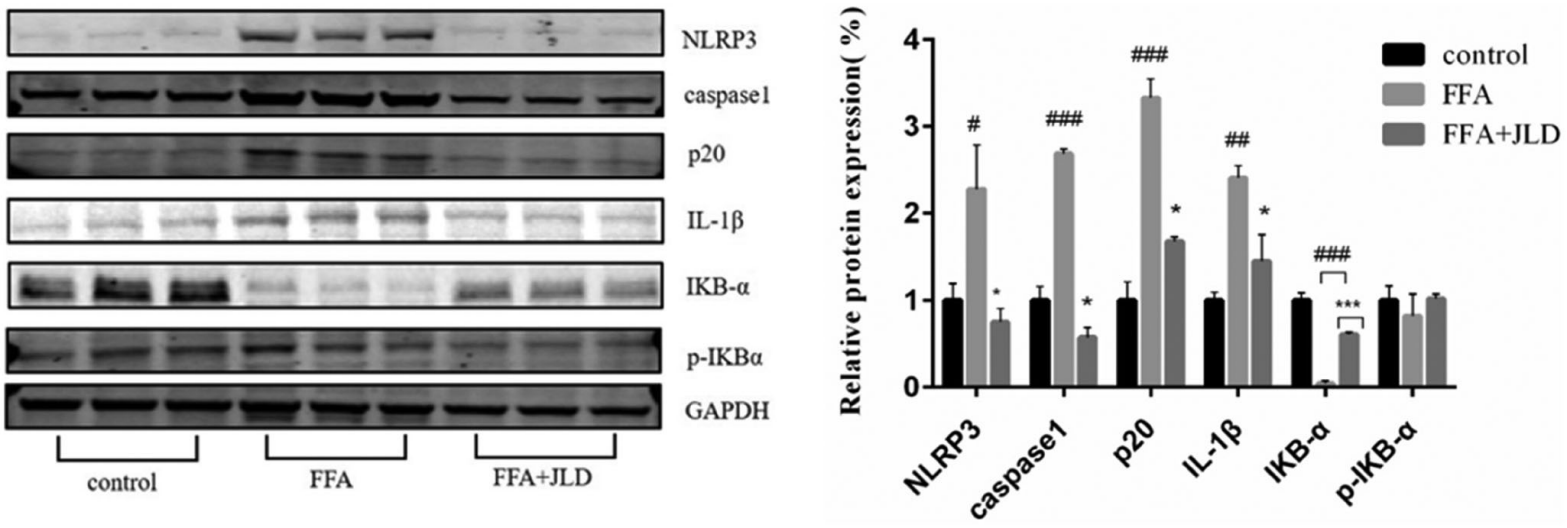
JLD protects HepG2 against pyroptosis induced by FFA. Expression of NLRP3, caspase 1, IL-1β, *p*-IKBα and IKBα protein in HepG2 cells. Data were normalised to GAPDH for western blot. #*p* < 0.05, ##*p* < 0.01, ###*p* < 0.001 vs. CON. *p < 0.05, **p < 0.01, ***p < 0.001 vs. FFA

At the molecular level, JLD decreased the protein expression of inflammatory markers such as IL-1β, NLRP3 and *p*-IKB-α, which was increased by FFA. Furthermore, JLD decreased the ratio of *p*-IKB-α/IKB-α. JLD decreased the protein levels of pro-caspase1 and p20 in FFA-treated cells by 78% and 49%, respectively. We found that JLD inhibited the activation process of inflammasome complexes and suppressed the activation of caspase1, which indicates that JLD reduced liver inflammation and tissue damage by inhibiting the pyroptosis pathway (Figure 5) (*p* < 0.05).

## Discussion

Generally, the TCM formula has multiple components and can interact with multiple cellular targets. JLD is a traditional Chinese medicine formula used to treat T2DM (Alberti et al. 2006; Lian et al. 2019). A total of 9 main phytochemical components in JLD has been identified, including salvianic acid A sodium, puerarin, salvianolic acid B, epimedin B, epimedin C, icariin, ginsenoside Rb1, ginsenoside Rc, and ginsenoside Rb2 (Wang et al. 2015). Clinical investigations reveal that JLD significantly reduced blood levels of fasting glucose, 2 h post-load glucose, and HbA1c levels in patients with T2D (Jin et al. 2020). Furthermore, JLD significantly improves glycemic control by stimulating insulin secretion and increasing insulin sensitivity (Lian et al. 2015; Tian et al. 2018). Pharmacologically, JLD increases the expression of genes involved in mitochondrial function and fat oxidation (Zang et al. 2015). Besides, JLD ameliorates the high-fat induced hyperglycaemia, hyperinsulinemia, and hyperlipidaemia in mice, which can be ascribed to its effect on the attenuation of hepatic oxidative stress and inhibition of intracellular lipid accumulation (Liu et al. 2015). JLD has been reported to exert potently hepatoprotective functions in HFD-induced metabolic disorders mice (Zhang et al. 2019). It is known that HFD-fed obese mice and FFA-treated hepatocytes showed obvious pyroptosis. (Zhong et al. 2018). All these results remind us the effect of JLD in maintaining hepatic cellular metabolism might arise from the capacity in regulating lipid metabolism and alleviate hepatocellular inflammation and damage.

C57BL/6J becomes obese, hyperglycaemia and insulin resistant and are susceptible to liver steatosis when fed a high-fat diet containing 60% Fat (Zhang et al. 2019). In this study, the C57BL/6J strain, subjected to high dietary fat for 16 weeks, showed increased efficiency of fat storage and manifested insulin resistance and liver steatosis. JLD improved HDF-induced obesity, metabolic disorders, and liver steatosis. These results were consistent with other studies in chronic HFD-fed rodent models (Zhong et al. 2018). An increased influx of saturated fatty acids to the liver leads to activation of the NLRP3 inflammasome (Csak et al. 2011), and the activation of NLRP3 inflammasomes triggers autocleavage and maturation of caspase-1 (Srinivasula et al. 2002). Caspase-1 mediates the transition of the immature pro-inflammatory cytokines pro-IL-1β and pro-IL-18 to their mature and active forms (Lamkanfi and Dixit 2012). A recent study has revealed that sulforaphane, a pharmacologic inhibitor of NLRP3 inflammasomes, prevents NAFLD in a mouse model subjected to a high-fat diet (HFD) (Yang et al. 2016), which suggests that regulating NLRP3 inflammasome activation is a potential therapeutic strategy for NAFLD. Our results demonstrated that JLD simultaneously suppressed the expression of the NLRP3 and caspase-1, which meant that JLD might not only inhibit the autocleavage and maturation of caspase-1 by NLRP3 inflammasomes but also transcriptionally regulate the protein synthesis of pro-IL-1β and pro-IL-18. Generally, pyroptosis is widely accepted as a caspase-1 dependent programmed cell death. To verify the anti-pyroptosis effect of JLD in liver tissues, the western blotting results revealed that JLD reduced protein production in both cleaved caspase-1 (*p*20), an active form of caspase-1, and IL-1β.

We demonstrated that JLD significantly reduced the HFD-induced liver steatosis and suppressed NLRP3 production *in vivo*. The HepG2 cell line possesses a well-differentiated hepatocyte function and has liver-specific metabolic effects. In this study, HepG2 cells were stimulated with FFA to mimic the HDF-induced liver steatosis *in vitro*. To explore the optimal dose of JLD, we exposed the HepG2 cells to JLD in a concentration gradient. JLD had no effects on the cell viability of HepG2 when the dose was lower than 400 mg/mL and had obvious cytotoxic effects at a concentration of 1600 mg/mL. Following the results of cell viability, we determined the concentration range (200 to 800 mg/mL) for the experiment of cell viability against the lipotoxicity induced by FFA. The optimal concentration of JLD for further study was determined at a concentration of 200 mg/mL. Compared with FFA alone, JLD significantly increased the cell viability of HepG2 and inhibited the intracellular TG contents at the concentration of 200 mg/mL. FFA-treated HepG2 cells were treated with or without JLD and stained with the lipid probe Nile red, which showed that JLD inhibited the lipid accumulation in HepG2 cells. As mentioned above, a high concentration of FFA can induce hepatocyte death via activating pyroptosis (Csak et al. 2011). To validate whether the protective actions of JLD on FFA-induced lipotoxicity to HepG2 cells was also due to the inhibition of pyroptosis. The results of western blotting showed that JLD could decrease the protein expression of NLRP3 and the pyroptosis execution molecular caspase-1, which was consistent with the anti-pyroptotic effects *in vivo*.

## Conclusion

The present study demonstrated that cell pyroptosis played a role in lipotoxicity-induced hepatocyte death. JLD not only improved the liver steatosis in the chronic HFD-fed mouse model but also antagonised the lipotoxicity-induced HepG2 hepatocytes pyroptosis. Thus, JLD is a potent NAFLD suppressor and an attractive therapeutic target for hepatic steatosis and related metabolic disorders. Although these results suggested that the beneficial effect of JLD on improvements in NAFLD was associated with attenuation of inflammatory stress, the therapeutic effects of JLD on NAFLD need further clinical validation.
